# 3D Printed Thermoelectric Polyurethane/Multiwalled Carbon Nanotube Nanocomposites: A Novel Approach towards the Fabrication of Flexible and Stretchable Organic Thermoelectrics

**DOI:** 10.3390/ma13122879

**Published:** 2020-06-26

**Authors:** Lazaros Tzounis, Markos Petousis, Sotirios Grammatikos, Nectarios Vidakis

**Affiliations:** 1 Composite and Smart Materials Laboratory (CSML), Department of Materials Science & Engineering, University of Ioannina, GR-45110 Ioannina, Greece; 2 Mechanical Engineering Department, Hellenic Mediterranean University, Estavromenos, 71004 Heraklion, Crete, Greece; markospetousis@hmu.gr; 3 Group of Sustainable Composites, Department of Manufacturing and Civil Engineering, Norwegian University of Science and Technology, 2815 Gjøvik, Norway; sotirios.grammatikos@ntnu.no

**Keywords:** conductive polymer composites (CPCs), three-dimensional (3D) printing, 3D printed thermoelectrics, polymer thermoelectrics, flexible and stretchable thermoelectrics

## Abstract

Three-dimensional (3D) printing of thermoelectric polymer nanocomposites is reported for the first time employing flexible, stretchable and electrically conductive 3D printable thermoplastic polyurethane (TPU)/multiwalled carbon nanotube (MWCNT) filaments. TPU/MWCNT conductive polymer composites (CPC) have been initially developed employing melt-mixing and extrusion processes. TPU pellets and two different types of MWCNTs, namely the NC-7000 MWCNTs (NC-MWCNT) and Long MWCNTs (L-MWCNT) were used to manufacture TPU/MWCNT nanocomposite filaments with 1.0, 2.5 and 5.0 wt.%. 3D printed thermoelectric TPU/MWCNT nanocomposites were fabricated through a fused deposition modelling (FDM) process. Raman and scanning electron microscopy (SEM) revealed the graphitic nature and morphological characteristics of CNTs. SEM and transmission electron microscopy (TEM) exhibited an excellent CNT nanodispersion in the TPU matrix. Tensile tests showed no significant deterioration of the moduli and strengths for the 3D printed samples compared to the nanocomposites prepared by compression moulding, indicating an excellent interlayer adhesion and mechanical performance of the 3D printed nanocomposites. Electrical and thermoelectric investigations showed that L-MWCNT exhibits 19.8 ± 0.2 µV/K Seebeck coefficient (*S*) and 8.4 × 10^3^ S/m electrical conductivity (*σ*), while TPU/L-MWCNT CPCs at 5.0 wt.% exhibited the highest thermoelectric performance (*σ* = 133.1 S/m, *S* = 19.8 ± 0.2 µV/K and *PF* = 0.04 μW/mK^2^) among TPU/CNT CPCs in the literature. All 3D printed samples exhibited an anisotropic electrical conductivity and the same Seebeck coefficient in the through- and cross-layer printing directions. TPU/MWCNT could act as excellent organic thermoelectric material towards 3D printed thermoelectric generators (TEGs) for potential large-scale energy harvesting applications.

## 1. Introduction

Three-dimensional (3D) printing has dramatically expanded over the last 20 years, while currently being a cutting-edge and one of the most widely used additive manufacturing (AM) technologies amongst others [1]. 3D printed parts and components due to variable mechanical and physicochemical properties that can be achieved could be employed in a wide range of applications [2]. A significant technological progress has been made for fused deposition modelling (FDM) printers and related machinery; however, a rapid growth has occurred lately towards the development of functional and novel printable thermoplastic materials, i.e., electrically conductive [3], magnetically active [4], flexible and stretchable [5], etc., filaments. Conductive polymer composites (CPCs) are known to offer various sensing functionalities, i.e., touch and pressure, temperature, chemical and/or electrochemical sensors [6]. Recently, electrically conductive filaments have been fabricated by employing carbon nanotubes (CNTs) and graphene as conductive filler incorporated in polylactide thermoplastic polymer matrix [7]. Moreover, CPC filaments have been reported for applications such as fabrication of FDM 3D printed strain sensors [8], resistors [9], biomedical sensors [10] and actuators [11].

Thermoelectric materials are by definition transducers of thermal to electrical energy being, thus capable of harvesting thermal energy and converting it into usable electricity. Specifically, thermoelectrics have the ability to generate an electric potential (known as thermoelectric voltage) upon being exposed to a temperature gradient, otherwise defined as the “Seebeck effect” [12]. It could be realised then that a high electrical conductivity (*σ*) combined with high Seebeck coefficient (*S =* ∆*V*/∆*T*) and low thermal conductivity (*κ*) are required for an efficient thermoelectric material. Traditional thermoelectric materials are typically based on low energy band gap inorganic semiconductors, e.g., Bi_2_Te_3_ [13], PbTe [14], GeTe [15], etc.; however, such materials do not allow potential applications for energy harvesting on a large-scale, since they consist of rare and high toxicity elements. The introduction of organic materials and especially CNTs as fillers in thermoplastic polymer matrices endowed CPCs with thermoelectric property and this is a field of research that has emerged over the last decade [16,17]. Namely, SWCNTs incorporated via solvent mixing in a non-conductive polycarbonate (PC) polymer matrix exhibited an electrical conductivity of approx. 1000 S/m and Seebeck coefficient *S* = +60 µV/K at 30 wt.% SWCNT loading [18].

CPCs based on polymeric matrices having electron-donating functional groups, as for instance Polyvinyl alcohol (PVA) and polyethyleneimine (PEI), showed n-type thermoelectric behaviour. Specifically, PEI/SWCNT nanocomposites with PEI functioning as n-doping to the incorporated SWCNTs resulted in Seebeck coefficients up to −21.5 µV/K [19]. Previous research from our group investigating the thermoelectric properties of melt-mixed polycarbonate (PC)/MWCNT CPCs at different MWCNT loadings, showed that higher CNT contents resulted in increased power factors (*PF* = *σ × S^2^* in *W*/mK^2^), which has been mainly explained by the increase of the electrical conductivity [20]. In another study, high-performance polyetherimide–SWCNT thermoplastic CPCs have been reported capable of operation up to 200 °C with electrical conductivity reaching 20 S/m and Seebeck coefficients +55 µV/K at 10 wt.% SWCNT loading [21]. Moreover, flexible thermoelectric devices based on serially interconnected p- and n-doped SWCNT thermocouples have been reported [22], as well as flexible semiconductor fibres for temperature sensing [23]. Recently, a detailed review article has elaborated the novelty of thermoelectric composites based on conducting polymer/carbon nanoparticle systems as promising green energy materials [24]. CPC filaments for FDM 3D printing and 3D objects have been reported so far [25]; however, 3D printed thermoelectric polymer nanocomposites that can be flexible and stretchable at the same time have not been reported yet.

In this study, TPU/MWCNT 3D printable, flexible, stretchable and thermoelectric filaments have been developed with two different types of commercially available MWCNTs, while 3D printed thermoelements were fabricated and fully characterised. Two different MWCNTs were utilised (NC-MWCNT and L-MWCNT) with significant differences in electrical conductivity, while both were introduced in a TPU matrix via a melt compounding/mixing process. Masterbatches of 5.0 wt.% TPU/NC-MWCNT and TPU/L-MWCNT were produced initially and in a next step reprocessed and diluted via melt mixing towards being finally extruded as filaments for the 3D printing process. SEM and transmission electron microscopy (TEM) exhibited the excellent nanocomposite microstructures (TPU/NC-MWCNT and TPU/L-MWCNT) with highly dispersed CNTs in the TPU matrix and without agglomerates that can affect the 3D printing process. All 3D printed samples exhibited an anisotropic electrical conductivity and same Seebeck coefficient since it is an inherent material property arising from the CNT filler. The 3D printed TPU/MWCNT thermoelements printed herein can be the building blocks for the fabrication of flexible and stretchable organic thermoelectric generators (TEGs).

## 2. Materials and Methods

### 2.1. Materials

TPU pellets and MWCNTs of two different types with different graphitization degrees and lengths were used as the constituents of the conductive polymer nanocomposites (CPNs) fabricated in this study. Namely, Elastollan 1185A grade of TPU polymer having a density of 1.12 g/cm^3^ and a shore hardness of 85A was received by BASF and utilised as the matrix of the nanocomposites. Commercially available MWCNTs, namely NC7000 and long MWCNTs—hereafter denoted as NC-MWCNTs and L-MWCNTs, respectively—were purchased from Nanocyl S.A. (Sambreville, Belgium). According to the supplier specifications, both MWCNT grades exhibited purity higher than 90%. The NC-MWCNTs is a highly conductive grade material with CNTs exhibiting average diameters of 9.5 nm and average lengths of 1.5 μm. NC-MWCNTs have been widely used elsewhere for melt-mixed conductive polymer composites (CPCs) [26,27,28]. The L-MWCNTs according to the supplier have slightly higher graphitization degree with CNTs of 9.5 nm in average diameter and consisting of longer CNTs with average lengths of 3.0 μm.

### 2.2. Fabrication of TPU/NC-MWCNT and TPU/L-MWCNT Filaments

TPU/5 wt.% MWCNT masterbatches (NC-MWCNT and L-MWCNT respectively) were produced initially using a small-scale twin-screw extruder (ZSC 20, Noris Plastic GmbH, Nürnberg, Germany). The melt temperature was 219–228 °C and screw rotational speed 300 rpm, while a screw length-to-diameter (L/D) ratio of 36 was employed for the melt mixing process. The TPU/CNT masterbatch preparation process parameters were followed as reported elsewhere [8]. All materials used for the masterbatch production were premixed in a glass vial and placed a in vacuum oven at 80 °C overnight, prior to the compounding process. TPU/MWCNT nanocomposites with MWCNT content of 1.0, 2.5 and 5.0 wt.% with diluted concentrations from the masterbatch were prepared then using a DSM Xplore Microcompounder (Geleen, Netherlands). The microcompounder used had a capacity of 15 cm^3^ with conical twin screws of 150 mm length and L/D ratio of 18. The following parameters were applied for the mixing/masterbatch dilution process: 230 °C as the mixing temperatures, of 5 min in total as the mixing time at a constant rotational speed of the screws set at 150 rpm. The extruded filaments were finally wound up utilising a Filabot spooler (Filabot, Vermont), while the extrusion parameters were also optimized in order to achieve a constant filament diameter at various MWCNT contents. This is a prerequisite for FDM printers that require consistent filament diameter for the calculation of the feed rate during the printing process. The extruded filament diameter was measured to be in all cases 1.68 ± 0.07 mm, which is adequate accuracy for consistent printing. This diameter was inserted in the slicer program to calculate the necessary feed rate that should be automatically used during the whole printing process. Neat TPU filament was prepared in the same manner.

### 2.3. Fused Deposition Modeling (FDM) of TPU/MWCNT Nanocomposites

A MakerBot Replicator 2 Experimental FDM printer (MakerBot, Brooklyn, NY, USA) and the MakerBot MakerWare software capable of processing STL files into thin slices to generate the G-code were used for 3D printing the TPU/MWCNT nanocomposite filaments. For optimum printability, the FDM printing parameters had to be initially optimized and finally the following printing conditions have been employed for all the printing processes in this study: nozzle temperature, 220 °C; print nozzle diameter, 0.8 mm; bed temperature, 60 °C; layer height, 0.20 mm; print infill, 100%; and print speed, 20 mm/s. The dimensions of the samples for the electrical conductivity and Seebeck coefficient measurements were 1.6 × 1.6 mm with a length of 100 mm. All 3D models for the 3D printing in this work have been designed using the 3D design Autodesk^®^ Fusion 360™ (Autodesk^®^, Inc., San Rafael, CA, USA) software, while being finally exported to Standard Tessellation Language (STL) files.

### 2.4. Characterization Techniques

A DXR Smart Raman instrument (Thermo Scientific, Waltham, MA, USA) was used to investigate the Raman spectroscopic responses of NC-MWCNT and L-MWCNT in this study. The spectra were acquired using a 532-nm laser line in the spectral region of 900–3000 cm^−1^ with a laser spot diameter of ~3 μm, while the spectral resolution achieved was 5 cm^−1^.

A JEOL JSM 6510 LV SEM/Oxford Instruments scanning electron microscope (SEM) (Jeol, Tokyo, Japan) operating at an accelerating voltage of 1.0 KV was employed for all the SEM investigations performed in this study. SEM was employed to study the morphology of NC-MWCNTs and L-MWCNTs in the form of bucky paper films, as well as for the TPU/MWCNT microstructural analysis to illustrate the nanotube dispersion in the TPU matrix. Cryo-fractured surfaces were prepared by immersing the samples in liquid nitrogen and broken perpendicular to the Y-printing direction. Transmission electron microscopy (TEM) (Libra 200 HR-TEM, Carl Zeiss AG, Oberkochen, Germany) analysis was performed in order to evaluate the MWCNT nanodispersion state in the TPU polymer matrix. Ultrathin sections of the polymer nanocomposites with a thickness of ~70 nm were prepared by means of cryo-ultramicrotomy using an ultramicrotome (Leica UC7, Leica Microsystems GmbH, Wetzlar, Germany), while more details could be found elsewhere [29,30]. The thin sections were placed on copper grids (300 mesh Cu, Agar), and further investigated with an FEI microscope operating at an acceleration voltage of 100 kV.

**Figure 1:**
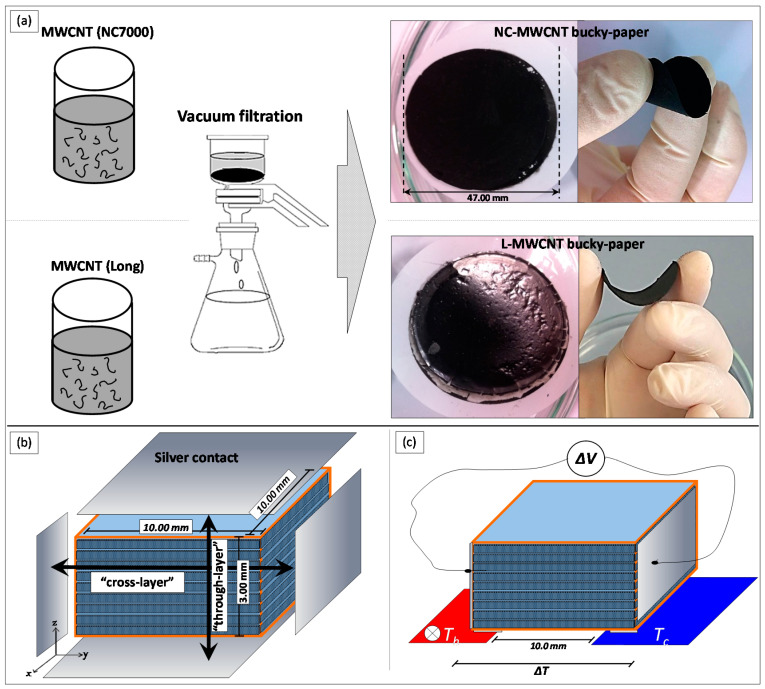
(**a**) Vacuum filtration process to fabricate MWCNT bucky paper films. (**b**) Schematic illustration of the cross- and through-layer electrical conductivity measurements for the different TPU/MWCNT FDM 3D printed nanocomposites, and (**c**) thermal gradient set-up used for the Seebeck coefficient measurements.

The electrical conductivity of NC-MWCNTs and L-MWCNTs in the form of rectangular-shaped films (30.0 mm length × 5.0 mm width with thickness: ~100 μm) was determined by measuring the room temperature sheet resistance (*Rs*) using a four-point probe system (Ossila Ltd., Sheffield, UK). NC-MWCNT and L-MWCNT bucky paper films were prepared initially employing a vacuum filtration apparatus using polycarbonate filter membranes (0.4 μm pore size) and 500 mL of CNT dispersions (0.3 mg/mL) with the help of SDBS surfactant (50% in relation to the CNT mass) and 30 min of bath sonication (Figure 1a). The filter cakes were cleaned with 20 mL of 30% hydrochloric acid (HCl) in order to remove the surfactant molecules, while 5 L of distilled water were filtered through the CNT films until reaching a neutral pH. The bucky papers were dried in a vacuum oven at 80 °C overnight and rectangular-shaped NC-MWCNT and L-MWCNT samples were cut for the sheet resistance derived electrical conductivity (*σ*) measurements (bucky paper film thickness ~100 μm). For the thermovoltage and electrical conductivity investigations of the TPU/MWCNT nanocomposites, the extruded filaments were cut into small pieces and thermally pressed under vacuum into circular-shaped samples with the following dimensions: 25.0 mm in diameter and 3.0 mm thickness. For all samples, the compression moulding temperature was 220 °C for 10 s and the pressure was set at 17.5 kN. The disks’ flat surfaces were coated then with low-temperature and fast curing silver paste (Agar scientific, Germany) to contact the samples, while the samples with specific dimensions resistance was measured using an Agilent Multimeter (Agilent 34401A6½, Agilent, Santa Clara, CA, USA) in order to derive further the electrical resistivity (*ρ*) and conductivity (*σ*). The through-thickness (through-layer) as well as in-plane (cross-layer) electrical conductivity of 3D printed samples was determined using square-shaped samples of 15.0 × 15.0 mm^2^ and 3.0 mm thickness. In both cases, the appropriate surfaces were coated with Ag paste, as illustrated in Figure 1b, and the conductivity was derived from a two-point probe electrical resistance measurements. This was performed in order to elucidate if there is any loss in “bulk” electrical conductivity resulting further into an anisotropic electrical conductivity behaviour due to the FDM printing process.

A self-made custom set-up was employed to determine the Seebeck coefficient for all conductive samples in this work. NC-MWCNT and L-MWCNT bucky paper films (30.0 mm length × 5.0 mm width with thickness: ~100 μm), as well as TPU/MWCNT nanocomposites of 1.0 wt.%, 2.5 wt.% and 5.0 wt.% from the compression moulding as well as FDM 3D printing process were measured (Figure 1c). Experimental details and full description of the method to contact the sample, measurement of the temperature gradient (∆*T*) the samples were exposed to, etc., have been previously reported [31,32]. In brief, the different samples were exposed to a temperature gradient stage where one block was maintained at room temperature (~298 K), while the other one was heated up to 373 K using a heating controller in 10 K steps. The generated thermoelectric voltage (Δ*V*) was measured with previously deposited metallic contacts using a digital multimeter (DMM) voltmeter (Agilent 34401A6½, Agilent, Santa Clara, CA, USA), capable of data logging to a PC. K-type thermocouples were placed onto the two blocks to continuously measure the temperature and precisely determine thus the temperature gradient, Δ*T*. The Seebeck coefficient was derived then from Δ*V* vs. Δ*T* curve’s slope by linear fitting. The Agilent 34401A6½ DMM that was employed in this work was operated in the measurement range of 100 mV which gives a maximum resolution of 100 nV in this range. Moreover, according to the manufacturer specifications, the measurement accuracy/error in this range is ±8.5 µV (1 Year DMM operating at 23 ± 5 °C; the temperature of the environment that all the measurements have been performed in our study).

For the thermal conductivity measurements performed at room temperature, samples with a disk geometry of 12.5 mm diameter and 2 mm thickness were required. The Netzsch LFA 447 Nano Flash (Netzsch equipment manufacturing GmbH, Selb, Germany) thermal conductivity commercial measurement set-up was used to study the thermal conductivity of all TPU/MWCNT nanocomposites. Circular plates of 12.5 mm and a thickness of 2 mm were prepared using hot-press in a similar manner as for the electrical conductivity measurements described above. The through-thickness (through-layer) thermal conductivity of 3D printed nanocomposites was measured also on 3D printed TPU/MWCNT nanocomposite samples with a diameter of 12.5 mm and a thickness of 2 mm.

Tensile test experiments of the nanocomposite samples prepared by compression moulding from the precursor filaments and 3D printed samples were carried out at room temperature using a Universal Testing Machine with a crosshead speed of 5 mm/s. As similarly reported in another study, rectangular and not dog-bone shaped samples (dimensions: 1.6 mm (width) × 1.6 mm (thickness) × 100 mm (length)) were tested in order to avoid possible stress concentration points that could be possibly created in the curvatures of a typical dog-bone sample with discrete lines [8].

## 3. Results and Discussion

### 3.1. Raman Analysis and Electrical Conductivity of NC-MWCNT and L-MWCNT

**Figure 2:**
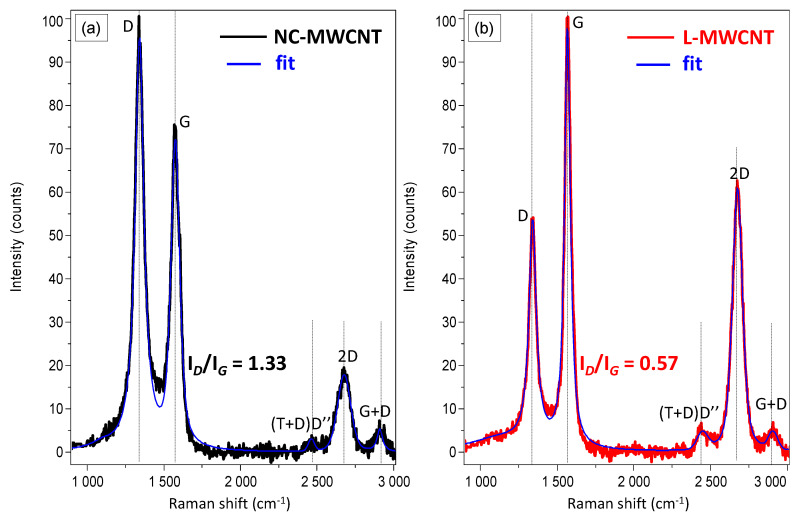
Raman spectra of (**a**) NC-MWCNT and (**b**) L-MWCNT showing characteristic bands of MWCNT material with different graphitization degree and number of defects of the CNT graphitic lattice.

Figure 2 shows the Raman spectrum of NC-MWCNT and L-MWCNT, respectively, in the spectral region of 900–3000 cm^−1^. In order to appropriately define the Lorentzian D- and G-peaks’ characteristics of the Raman spectra, a curve fitting analysis was performed. Namely, the G- and D-band peaks were centred at 1565 and 1345 cm^−1^ and these signals are characteristic of CNTs [33]. It is worth mentioning that radial breathing mode (RBM) peaks at, i.e., 100–300 cm^−1^, were absent, indicating that the CNTs were of multiwalled (MWCNTs) type rather than single-wall carbon nanotubes (SWCNTs). Additionally, the detection of the 2D band also confirms that the nanotubes are of MWCNT type [16]. In the spectra, two distinct spectral regions can be observed: one at 1000 and 2000 cm^−1^ and the second one between 2300 and 3000 cm^−1^. The first one is governed by two broad bands, namely the G-band (G stands for “graphitic”) between 1500 and 1600 cm^−1^, and the D-band (D stands for “defects” or disorder) at 1300–1400 cm^−1^, which results from a disorder-induced double resonant process due to the breakdown of the usual wave vector selection rule (A_1g_ symmetry) [34]. In the second-order region, three main bands between 2300 and 3000 cm^−1^ could be observed, i.e., the (T + D)D′′ (2450 cm^−1^), 2D (2675 cm^−1^) and the G + D (2900 cm^−1^), which are assigned to overtones and combinations of the bands arising from the first order region. The graphitization and degree of crystallinity of CNTs have been several times correlated to the relative intensity ratio of the corresponding D and G-bands (*I*_D_/*I*_G_) as a well-established indicator [35]. Specifically, the D and G band intensity ratio (*I_D_/I_G_*) decreases from 1.33 for NC-MWCNT to 0.57 for L-MWCNT. This can be explained by a larger number of defects and existing sp^3^-hybridized carbons in the nanotube framework, which may affect further the MWCNT charge carrier transport properties responsible for both the electrical conductivity and thermoelectric properties. The inherent electrical conductivity of NC-MWCNT and L-MWCNT measured with a four-point probe method on bucky paper films revealed an electrical conductivity of 1.6 × 10^3^ S/m for NC-MWCNT and 8.4 × 10^3^ S/m for L-MWCNT. This is more precisely attributed to the higher graphitic nature of L-MWCNT compared to NC-MWCNT shown by the Raman analysis.

### 3.2. Morphological Analysis of NC-MWCNT and L-MWCNT

**Figure 3:**
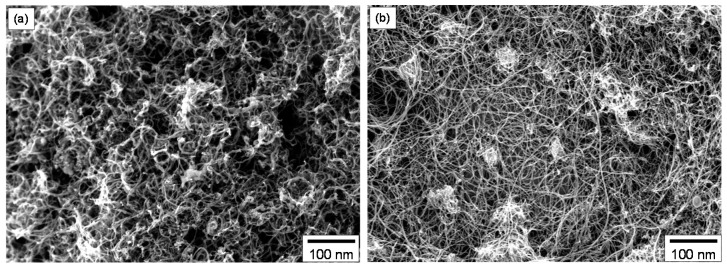
SEM images of bucky paper films consisting of (**a**) NC-MWCNT and (**b**) L-MWCNT.

SEM analysis showed distinct differences of the NC-MWCNT (Figure 3a) as compared to the L-MWCNT (Figure 3b), investigated by the corresponding CNT bucky paper films. In both cases, highly entangled CNTs can be observed characteristic of bucky paper film morphology. The NC-MWCNT exhibit in general shorter lengths as well as an apparent tendency to curve in their main axis. On the other hand, the L-MWCNT exhibit quite high CNT lengths, which directly affects the CNT aspect ratio known to be an important parameter for the CNT electrical transport properties as well as affecting the electrical percolation threshold and the induced bulk conductivity polymer/CNT nanocomposites.

### 3.3. Microstructure Investigations of the FDM 3D Printed TPU/MWCNT Nanocomposites

**Figure 4:**
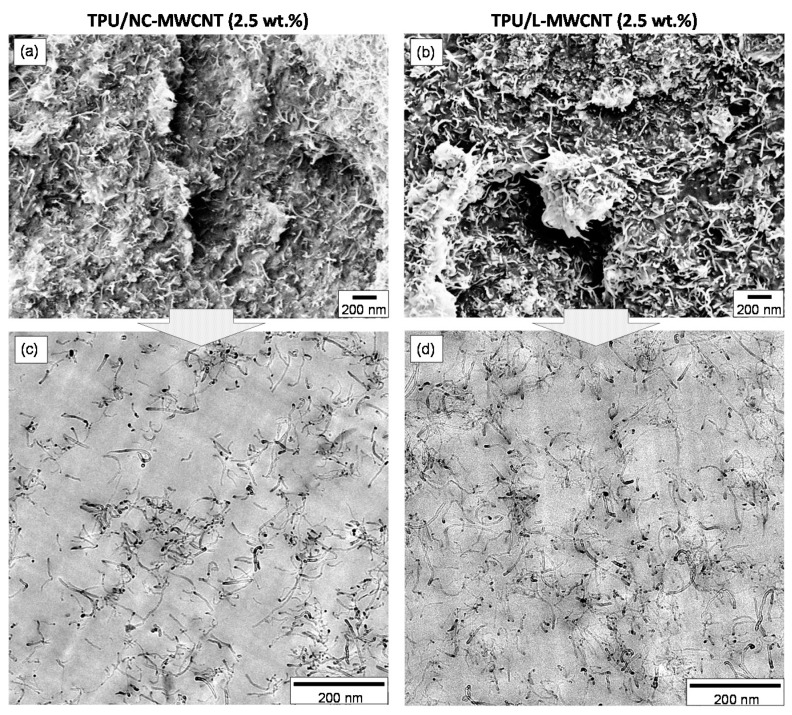
(**a**,**b**) SEM images of the 3D printed TPU/NC-MWCNT (2.5 wt.%) and TPU/L-MWCNT (2.5 wt.%) fractured surfaces, respectively. (**c**) TEM images of the TPU/NC-MWCNT (2.5 wt.%) and (**d**) TPU/L-MWCNT (2.5 wt.%) nanocomposites showing the achieved CNT nanodispersion in the TPU matrix.

The SEM and TEM images of 3D printed TPU/MWCNT nanocomposites of 2.5 wt.% MWCNT content could be seen in Figure 4. The SEM images were acquired from cryo-fractured surfaces of FDM 3D printed samples broken perpendicular to the Y-printing direction, while crossections for the TEM analysis were also obtained from sample’s broken “block-surface” perpendicular to the Y-printing direction. Figure 4a shows the SEM surface morphology of TPU/NC-MWCNT, while Figure 4b shows the morphology of TPU/L-MWCNT. In both cases, a homogeneous distribution of CNTs in the TPU polymer matrix could be observed with quite rough surfaces and visible CNTs due to the fracture process. In general the TPU/L-MWCNT exhibits much higher roughness compared to TPU/NC-MWCNT, possibly due to the longer CNT lengths that some are pulled out from the polymer matrix upon the destructive cryofracture process. Moreover, a highly entangled CNT network could be observed endowing the electrical and thermoelectric properties to the TPU/MWCNT nanocomposites. Figure 4c illustrates the TEM image of TPU/NC-MWCNT nanocomposite, while Figure 4d shows that of TPU/L-MWCNT. The TEM images reveal the MWCNT nanodispersion status, while both NC-MWCNTs and L-MWCNTs do not form any primary as well as secondary agglomerates. MWCNTs have been sufficiently disentangled in both cases with single dispersed nanotubes observed within the TPU matrix creating a high quality of geometrically percolated CNT network. This is attributed to the excessive infiltration of the CNT clusters by the TPU polymer melt and the shear forces during melt mixing resulting in highly dispersed CNT nanofillers, desired for the optimum electrical conductivity of the final nanocomposites. The high quality and dispersion of MWCNTs within the TPU polymer matrix would allow enhanced electrical conductivity and mechanical properties of the final nanocomposites [36]. Furthermore, it is critical that a high quality of CNT dispersion has been observed within the TPU/MWCNT nanocomposites, since the existence of micro-aggregates can cause nozzle clogging and affect the final 3D printed object’s quality.

### 3.4. Mechanical Properties

**Figure 5:**
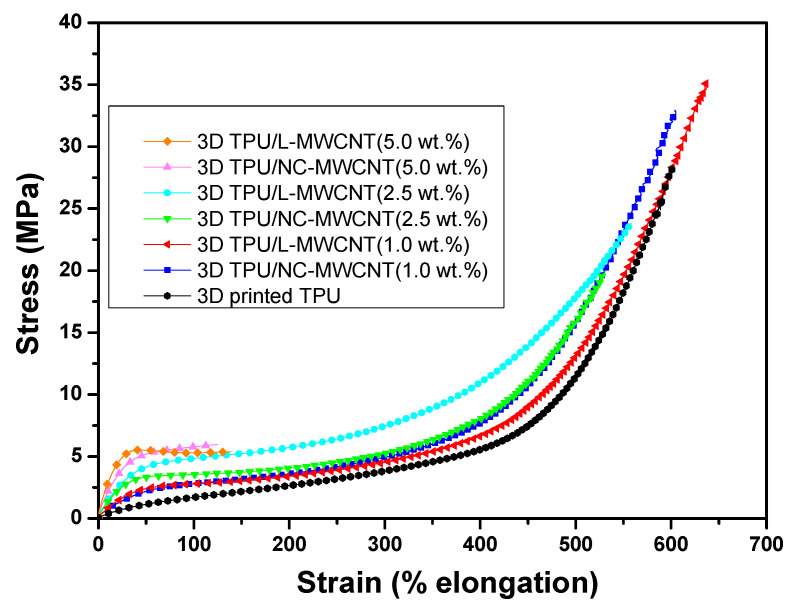
Stress–strain curves of all 3D printed samples, namely TPU, TPU/NC-MWCNT and TPU/L-MWCNT nanocomposites at 1.0, 2.5 and 5.0 wt.% MWCNT loadings.

Table 1 summarizes the measured modulus, tensile strength and elongation at break of the extruded neat TPU, TPU/NC-MWCNT and TPU/L-MWCNT at 1.0 wt.%, 2.5 wt.% and 5.0 wt.% loadings, as well as 3D printed TPU, TPU/NC-MWCNT and TPU/L-MWCNT with the same filler loading. For all samples, the initial 10% strain region was used in order to calculate the respective moduli, where the stress–strain relationship was linear. In general, the strength of both the TPU-extruded filaments and the FDM 3D sprinted ones increased with the addition of MWCNTs, being in good agreement with a review that can be found in the literature [37]. On the other hand, the modulus has been slightly decreased after 3D printing. On average, a decrease of ~11.9% has been calculated comparing the moduli of the filament derived compression moulded samples with the corresponding FDM 3D printed ones. This is obviously superior to what has been already reported in the literature for 3D printed ABS samples compared to bulk ABS [3]. Another important finding to point out is a slight decrease in the strength of all the fabricated FDM 3D printed samples, compared to their respective filament counterparts. This is due to the FDM additive manufacturing 3D printing process resulting into small interlayer voids, as well as plausibly interfacial weak and/or imperfect interlayer bonding as has been reported in several studies [8]. It is also worth mentioning that the 3D printed TPU/NC-MWCNT and TPU/L-MWCNT nanocomposites with the maximum MWCNT content (5.0 wt.%) could be strained only up to 118% and 129%, respectively. This low-strain failure can be ascribed to the weak adhesion between the 3D sample layers or to existing CNT micro-aggregates in such high filler loading that may act as “defects” and/or stress concentration points within the material decreasing significantly the % elongation at break and tensile strength. Subsequently, (i) more possible defects and (ii) weak interfacial strength of the interlayer bonds could act as stress concentration locations, causing the 5 wt.% filled samples to fail at lower strains. Figure 5 shows, representatively, the stress–strain curves of all 3D printed samples, namely the TPU, TPU/NC-MWCNT and TPU/L-MWCNT nanocomposites at 1.0 wt.%, 2.5 wt.% and 5.0 wt.% MWCNT loadings. On one hand, the TPU/L-MWCNT nanocomposites depict a more prominent increase of the moduli as well as of the tensile strength for all the MWCNT filler loadings compared to the TPU/NC-MWCNT (short CNTs). This is attributed to the higher aspect ratio and length of L-MWCNTs as well as the higher graphitic nature possible affecting the inherent mechanical properties of CNTs. On the other hand, the tensile strength of all TPU/MWCNT nanocomposites is increased with the addition of 1.0 wt.% MWCNTs, while showing a decrease for 2.5 and 5.0 wt.% of MWCNT loadings.

### 3.5. Electrical Conductivity of TPU/MWCNT Nanocomposites

**Figure 6:**
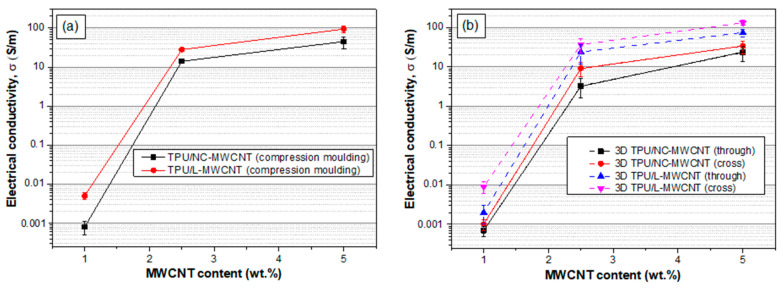
(**a**) Electrical conductivity of TPU/NC-MWCNT and TPU/L-MWCNT thermal-pressed nanocomposites at 1.0, 2.5 and 5.0 wt.% MWCNT content (at “in-plane” direction of the compression moulded films). (**b**) Electrical conductivity of the 3D printed TPU/MWCNT nanocomposites at 1.0, 2.5 and 5.0 wt.% MWCNT contents at “through layer” and “cross-layer” printing directions.

Figure 6a shows the in-plane electrical conductivity of TPU/NC-MWCNT and TPU/L-MWCNT thermal-pressed nanocomposites at 1.0, 2.5 and 5.0 wt.% MWCNT content. The highest conductivities were determined for the 5.0 wt.% TPU/MWCNT nanocomposites, namely 45.2 S/m (TPU/NC-MWCNT) and 94.1 S/m (TPU/L-MWCNT). It should be noted that the electrical conductivities of NC-MWCNT was 1.6 × 10^3^ S/m, while for the L-MWCNT 8.4 × 10^3^ S/m. This reveals that the resulting TPU/MWCNT nanocomposites reached two orders of magnitude lower electrical conductivity values at 5.0 wt.% MWCNT content, as compared to the inherent electrical conductivity of the MWCNTs used in each case as the electrically conductive filer. The higher conductivity of TPU/L-MWCNT at all MWCNT contents may be attributed to two factors: (i) due to the inherently higher electrical conductivity of L-MWCNTs and (ii) due to the aspect ratio of the filler that has been reported to strongly affect the percolation threshold as well as the electrical conductivity of the resulting CPCs [38,39]. Figure 6b shows the electrical conductivity of the 3D printed TPU/MWCNT nanocomposites at 1.0, 2.5 and 5.0 wt.% MWCNT contents at two different measurement directions, namely the “through layer” or through thickness as we as the “cross-layer” or in-plane of the 3D printed object. As it can be observed, the electrical conductivity values were in the same range as for the pressed ones showing that the conductivity of TPU/MWCNT nanocomposites was well maintained in both directions after the FDM printing. However, the scattering of the values, indicated by the corresponding standard deviations, was a bit higher compared to the compression moulded ones due to possibly more inhomogeneities of the 3D printed samples. It is also interesting to mention that for all 3D printed TPU/NC-MWCNT and TPU/L-MWCNT nanocomposites at different MWCNT contents, the cross layer conductivity values were a bit higher than the through layer ones most likely attributed to the FDM 3D printing process endowing thus an “anisotropic” character of the electrical properties of the final nanocomposites. More precisely, a plausible explanation for the slightly higher electrical conductivity in the cross layer direction could be that the printing process induces some extent of CNT orientation in the print deposition direction, resulting thus in a slightly better “bulk” electrical conductivity. This finding has been also observed and reported in a previous study for 3D printed CPCs [8].

### 3.6. Seebeck Coefficient (S), Power Factor (PF) and Thermoelectric Figure of Merit (ZT) of TPU/MWCNT Nanocomposites

**Figure 7:**
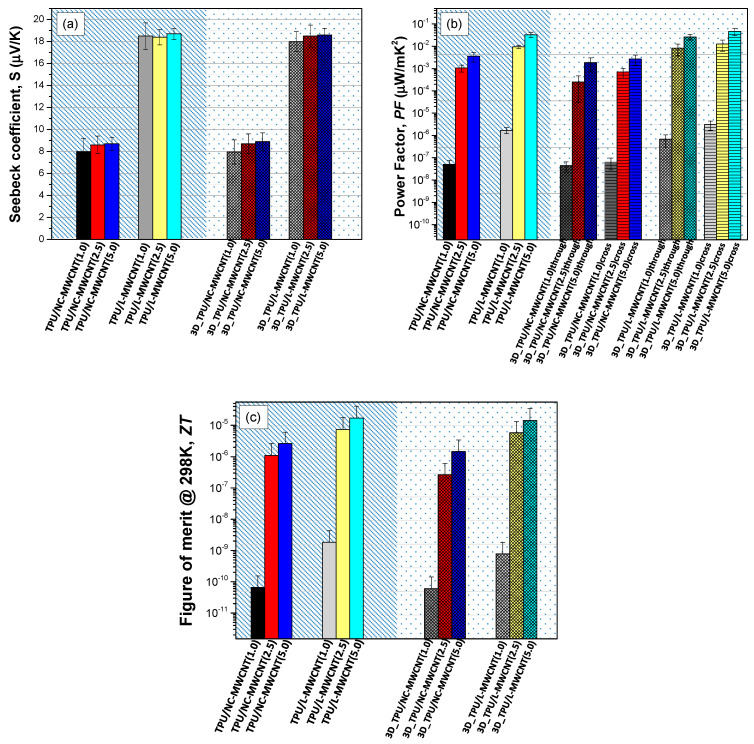
(**a**) Seebeck coefficient of TPU/NC-MWCNT and TPU/L-MWCNT nanocomposites prepared by compression moulding of the corresponding precursor filaments, and (**b**) *PF* values of the compression moulded as well as the 3D printed TPU/MWCNT nanocomposites at 1.0, 2.5 and 5.0 wt.% MWCNT content, considering the through- and cross-layer-measured electrical conductivities (Seebeck coefficient arising only from the CNT material is the same in all measured directions since it is an inherent property of the NW- and L-MWCNTs, respectively), (**c**) Figure of merit (*ZT*) of the compression moulded, as well as the 3D printed TPU/MWCNT nanocomposites (1.0, 2.5 and 5.0 wt.% MWCNT content).

Figure 7 presents the Seebeck coefficient, power factor (*PF*) and thermoelectric figure of the merit *(ZT)*-derived values of the compression moulded as well as the 3D printed TPU/MWCNT nanocomposites at 1.0, 2.5 and 5.0 wt.% MWCNT content. Specifically, Figure 7a demonstrates the Seebeck coefficient of TPU/NC-MWCNT and TPU/L-MWCNT nanocomposites prepared by compression moulding of the corresponding precursor filaments. The Seebeck coefficient for both pressed and 3D printed TPU/NC-MWCNT nanocomposites was in the range of 8.0–8.5 µV/K with a tendency to slightly increase from the 1.0 to 5.0 wt.% reaching ~8.5 µV/K, as the sample showed a higher CNT content and getting far from the percolation threshold. On the other hand, the Seebeck coefficient for both pressed and 3D printed TPU/L-MWCNT nanocomposites was much higher, namely in the range of 18.0–18.5 µV/K. The Seebeck coefficient of the NC-MWCNT and L-MWCNT measured in the bucky paper form were 9.2 ± 0.3 and 19.8 ± 0.2 µV/K, respectively. It should be noted herein that the thermoelectric property which arises from the CNT material is endowed to the respective TPU/MWCNT nanocomposites; while it is being preserved through all the different melt-mixing, compression moulding and 3D printing processes.

Figure 7b demonstrates the *PF* values of the compression moulded as well as the 3D printed TPU/MWCNT nanocomposites at 1.0, 2.5 and 5.0 wt.% MWCNT content, considering the through layer and cross-layer-measured electrical conductivities. It can be seen that the *PF* values increase in general by increasing the CNT content in all CPCs, while this is due only to the increase in the nanocomposite’s electrical conductivity at higher CNT loadings. Moreover, the *PF* values are higher for the TPU/L-MWCNT nanocomposites compared to the TPU/NC-MWCNT ones, due to both the higher electrical conductivity and Seebeck coefficient values. An interesting point also to be figured out is the anisotropic thermoelectric behaviour of the 3D printed TPU/NC-MWCNT and TPU/L-MWCNT nanocomposites at different MWCNT contents for the through- and cross-layer directions. For both TPU/NC-MWCNT and TPU/L-MWCNT nanocomposites, the derived *PF* values are slightly higher for the cross-layer directions attributed only to the higher electrical conductivity in cross-layer that has been determined and discussed previously. Namely, the highest *PF* value was found for the 3D printed TPU/L-MWCNT nanocomposite at 5.0 wt.% CNT content (*PF* = 0.02 μW/mK^2^ and 0.04 μW/mK^2^ for through layer and cross-layer direction, respectively).

CNT-based polymer nanocomposites with non-conductive engineered polymers (thermoplastics, thermosets, elastomer), as well as with conductive conjugated polymer matrices, have been reported several times until now, while TEG devices have been fabricated also using serially interconnected polymer nanocomposite thermocouples [40]. Namely, solvent-mixed single-walled carbon nanotubes (SWCNTs) with relatively high filler loadings (>>50 wt.%) in poly (3,4-ethylenedioxythiophene): poly (styrene sulfonate) (PEDOT:PSS) have reached the highest ever reported *PF* value of 140 μW/mK^2^ amongst polymer/CNT nanocomposites [41]. However, the low thermal stability and hygroscopic nature of PEDOT:PSS is an obstacle to various applications. Engineered and high temperature resistance thermoplastic polyetherimide (PEI)/SWCNT nanocomposites prepared by solvent mixing have been previously reported by our group, reaching a *PF* of 3.7 × 10^−2^ μW/mK^2^ at 10.0 vol% of SWCNTs [21]. Polycarbonate/MWCNT melt-mixed nanocomposites have been also reported with *PF* of ~8 × 10^−4^ μW/mK^2^ at 2.5 wt.% MWCNT loading [42]. In another study, Polypropylene-based melt-mixed composites with fixed SWCNT (2 wt.%) and copper oxide (5 wt.%) showed that using a low-molecular weight polyethylene glycol (PEG) during melt mixing as a process additive, p-type composites switched into n-type while the highest *PF* value achieved was in the range of ~5 × 10^−3^ μW/mK^2^ [40]. To the best of the authors’ knowledge, it could be realised that, in this research work, the highest ever reported value of *PF* has been achieved for melt-mixed and 3D printable/processed TPU/L-MWCNT stretchable nanocomposites at 5.0 wt.% of MWCNT content (0.02 and 0.04 μW/mK^2^ for through- and cross-layer directions). The *PF* values are slightly lower than our previously reported solvent mixed PEI/SWCNT nanocomposites at 10 vol.% SWCNT content mentioned above (*PF* = 3.7 × 10^−2^ μW/mK^2^) [21].

Figure 7c summarizes the dimensionless thermoelectric figure of merit (*ZT*) of the compression-moulded, as well as the 3D printed TPU/MWCNT nanocomposites at 1.0, 2.5 and 5.0 wt.% MWCNT content, as a well-known measure to determine the materials’ thermoelectric efficiency (*ZT;* $ZT=\frac{\sigma \times S^{2}}{\kappa }T=\frac{PF}{\kappa }T\;$, *κ* is the thermal conductivity, *PF* the power factor, and *T* the absolute temperature) [26]. For the calculation of the 3D printed nanocomposite’s *ZT*, it is worth mentioning that the “through layer” *PF* values were used to derive the corresponding *ZT* presented values. Specifically, the *ZT* values increase with increased CNT content in all CPCs. Moreover, the *ZT* values are higher for the TPU/L-MWCNT nanocomposites compared to the TPU/NC-MWCNT ones, due to higher *PF* values with slight variations in the thermal conductivity values (Table 2). Namely, the highest *ZT* values were found for the TPU/L-MWCNT nanocomposite at 5.0 wt.% CNT content (*ZT* = 1.42 × 10^−5^ and 1.69 × 10^−5^ for the compression moulding and the 3D printed fabricated nanocomposite, respectively).

The thermal conductivity of the TPU/CNT nanocomposites is shown in Table 2. As it can be observed, all samples exhibited relatively low thermal conductivity values, i.e., in the range of 0.22–0.55 W/mK [43] with slight increase with the increased CNT filler loading. It is worth mentioning that the low thermal conductivity of the nanocomposites is an important characteristic for the utilization of the TPU/MWCNT CPC nanocomposites as efficient thermoelectric materials, due to their ability to sustain a temperature difference upon being exposed to a temperature gradient (∆*T*). Therefore, the sustained temperature gradient within the material is the driving force for the thermoelectrically generated charge carriers. The thermal conductivity values are in accordance with the previously reported melt-mixed PC/MWCNT CPC materials, where it has been shown that the thermal conductivity remains below 0.6 W/m K^−1^ even at 15 wt.% of MWCNT filler content. Overall, it should be made clear that the *ZT* for such CPC nanocomposites has to be significantly increased in the future, i.e., by at least two orders of magnitude, in order to enable the commercialization of the proposed materials and fabrication of thermoelectric generator (TEG) power-generating devices made thereof. It is expected that melt-mixed thermoplastics with (i) higher CNT loadings, and (ii) the use of differently functionalized/doped CNTs, focusing mainly on SWCNTs that are more conductive materials, could be promising approaches to increase the electrical conductivity, as well as the Seebeck coefficient of the resulting CPCs towards highly efficient polymer/CNT organic thermoelectrics.

## 4. Conclusions

3D printed thermoelectric polymer nanocomposite materials that can be also flexible and stretchable have been fabricated for the first time and fully characterised in the study at hand. An optimised process of combined melt-mixing compounding and extrusion was developed for the fabrication of 3D printable TPU/MWCNT nanocomposite filaments with 1.0, 2.5 and 5.0 wt.% CNT contents, while two different types of commercially available CNTs with significant differences in morphology, electrical and thermoelectric properties have been utilised. The 3D printed TPU/MWCNT nanocomposite samples exhibited an anisotropic electrical conductivity in the through- and cross-layer printing direction, while no difference in the Seebeck coefficient since it is an inherent material property arising from the CNT type. The 3D printed TPU/MWCNT thermoelements can be the building blocks for the fabrication of flexible and stretchable organic thermoelectric generators (TEGs) by 3D printing FDM technology combined with an ink-jet printer head for the fabrication of the metallic contacts required as junctions for the serial interconnection of the thermoelements. The 3D printed thermoelectric TPU/MWCNT materials could open new avenues towards large-scale thermal energy-harvesting applications, i.e., wearables, stretchable 3D shaped harvesters, etc., where complex 3D architectures and customizability are required.

## Figures and Tables

**Table 1:** Modulus, tensile strength and elongation at break of the extruded TPU, TPU/NC-MWCNT and TPU/L-MWCNT as well as 3D printed TPU, TPU/NC-MWCNT and TPU/L-MWCNT nanocomposites.

Sample.	E-Modulus (MPa)	Tensile Strength (MPa)	Elongation at Break (%)
TPU	10.28 ± 1.22	33.78 ± 1.34	612
3D printed TPU	7.96 ± 1.67	32.12 ± 1.16	601
TPU/NC-MWCNT (1.0 wt.%)	17.56 ± 1.25	28.54 ± 1.84	632
3D TPU/NC-MWCNT (1.0 wt.%)	14.85 ± 1.13	27.86 ± 1.92	625
TPU/NC-MWCNT (2.5 wt.%)	22.32 ± 2.33	22.65 ± 1.32	575
3D TPU/NC-MWCNT (2.5 wt.%)	20.08 ± 2.84	21.08 ± 1.97	530
TPU/NC-MWCNT (5.0 wt.%)	25.01 ± 2.55	9.55 ± 1.12	154
3D TPU/NC-MWCNT (5.0 wt.%)	24.13 ± 2.98	5.85 ± 2.02	118
TPU/L-MWCNT (1.0 wt.%)	19.84 ± 1.12	29.35 ± 1.74	644
3D TPU/L-MWCNT (1.0 wt.%)	16.54 ± 1.26	28.13 ± 1.45	637
TPU/L-MWCNT (2.5 wt.%)	24.52 ± 2.86	24.86 ± 1.56	602
3D TPU/L-MWCNT (2.5 wt.%)	22.34 ± 2.49	23.54 ± 1.23	558
TPU/L-MWCNT (5.0 wt.%)	28.56 ± 3.95	10.45 ± 0.98	161
3D TPU/L-MWCNT (5.0 wt.%)	26.67 ± 3.44	6.48 ± 1.14	129

**Table 2:** Thermal conductivity of the extruded TPU, TPU/NC-MWCNT and TPU/L-MWCNT as well as 3D printed TPU, TPU/NC-MWCNT and TPU/L-MWCNT nanocomposites (at 1.0, 2.5 and 5.0 wt.% filler content).

Sample	Thermal Conductivity, *κ* (W/m K)
TPU	0.20 ± 0.01
3D printed TPU	0.19 ± 0.02
TPU/NC-MWCNT (1.0 wt.%)	0.23 ± 0.02
3D TPU/NC-MWCNT (1.0 wt.%)	0.22 ± 0.01
TPU/NC-MWCNT (2.5 wt.%)	0.29 ± 0.02
3D TPU/NC-MWCNT (2.5 wt.%)	0.28 ± 0.01
TPU/NC-MWCNT (5.0 wt.%)	0.41 ± 0.04
3D TPU/NC-MWCNT (5.0 wt.%)	0.38 ± 0.02
TPU/L-MWCNT (1.0 wt.%)	0.28 ± 0.02
3D TPU/L-MWCNT (1.0 wt.%)	0.26 ± 0.01
TPU/L-MWCNT (2.5 wt.%)	0.39 ± 0.03
3D TPU/L-MWCNT (2.5 wt.%)	0.42 ± 0.02
TPU/L-MWCNT (5.0 wt.%)	0.58 ± 0.04
3D TPU/L-MWCNT (5.0 wt.%)	0.55 ± 0.02
